# pH-Tuning the State-of-the-Art TELSAM Crystallization Chaperone

**DOI:** 10.1063/4.0001135

**Published:** 2025-10-27

**Authors:** Miles J Bradford

**Affiliations:** Brigham Young University, Provo, UT, USA

## Abstract

TELSAM fusion proteins allow guest proteins with weaker crystal contacts to form stable crystals, which is a powerful weapon against recalcitrant target proteins. However, this tendency can sometimes lead to premature and rapid crystal nucleation leading to precipitation during purification. With new adjustments in the TELSAM module and careful testing of solubility and turbidity in various model TELSAM fusion proteins, we report a significant, tunable increase in TELSAM solubility. This improvement is a direct result of the simple mutation of a leucine into a glutamate residue, which confers pH responsiveness to TELSAM by allowing its nucleation to be greatly slowed under high pH. This poster presents the data from the turbidity and precipitation assays, highlighting how this improvement in TELSAM will continue to advance our research into proteins and other biological molecules in the future.

